# Comparison of Externally Transferred and Self-Recruited Patients with Hip and Knee Revision Arthroplasty at a Certified Maximum-Care Arthroplasty Center (ACmax)

**DOI:** 10.3390/healthcare12181869

**Published:** 2024-09-18

**Authors:** Anika Marit Eismann, Annett Klinder, Wolfram Mittelmeier, Martina Rohde-Lindner, Katrin Osmanski-Zenk

**Affiliations:** Orthopedic Clinic and Policlinic, Rostock University Medical Center, D-18057 Rostock, Germany; anika.eismann@uni-rostock.de (A.M.E.); annett.klinder@med.uni-rostock.de (A.K.); wolfram.mittelmeier@med.uni-rostock.de (W.M.); martina.rohde-lindner@med.uni-rostock.de (M.R.-L.)

**Keywords:** EndoCert, Patient Clinical Complexity Level, Case Mix Index, comorbidities, septic interventions

## Abstract

Background/Objectives: According to the guidelines of the EndoCert initiative, certified maximum-care arthroplasty centers (ACmax) are obliged to admit patients from certified arthroplasty centers (AC) if these patients need to be transferred to the more specialized ACmax due to difficult replacement and revision procedures as well as after complications in primary care that are beyond the expertise of the smaller centers. This study investigated whether the cohort of transferred patients differed from the patients directly recruited at the ACmax for factors such as severity of diagnosis, comorbidities or outcome. The aim was to determine whether transferred patients increased the resource requirements for the ACmax. Methods: A total of 136 patients were included in the retrospective study and analyzed in terms of case severity, length of hospital stays (LOS), Diagnosis-Related Group charges, readmission rate and concomitant diseases. All patients were followed for up to 12 months after the initial hospital stay. Results: There were significant differences between the groups of transferred and self-recruited patients. For example, transferred patients had a higher Patient Clinical Complexity Level (PCCL). Similarly, the increased Case Mix Index (CMI) of transferred patients indicated more intensive care during the inpatient stay. The higher values for the comorbidity indices also supported these results. This had an impact on the LOS and overall costs, too. The differences between the groups were also reflected by adverse events during the one-year follow-up. The higher percentage of patients with septic revisions, whose treatment is especially demanding, among transferred patients aggravated the differences even further. Thus, transferred patients were associated with increased resource requirements for the ACmax. Conclusions: While it serves patients’ safety to transfer them to an ACmax with specialized expertise and greater structural quality, the care of transferred patients ties up considerable resources at the ACmax that might only be insufficiently reimbursed by the generalized tariffs.

## 1. Introduction

Since 2012, the EndoCert initiative, a German initiative to improve arthroplasty care, has regulated structural and surgeon-related processes to ensure a high quality of care [1]. Structurally, the initiative distinguishes between certified arthroplasty centers (AC) and certified maximum-care arthroplasty centers (ACmax). Some of the distinguishing features of an ACmax are the standardized structures and processes for carrying out even difficult replacement or revision procedures as well as dealing with severe complications [2]. Certified arthroplasty centers (AC), on the other hand, mainly provide primary arthroplasty care and cooperate with an ACmax for the more challenging cases. As part of the EndoCert initiative, ACmax are obliged to accept patients referred by ACs [3] in addition to their own planned surgical procedures and interventions. For specialized clinics in particular, such as ACmax, this is thought to result in an increased need for personnel, materials and time.

Our ACmax entered into cooperation with several ACs since its initial certification in 2012. Thus, our ACmax accepts transferred patients from surrounding hospitals, including non-certified facilities. The majority of transferred patients suffer from periprosthetic infections with an increased risk of sepsis.

This study used a retrospective data analysis to investigate the differences between a patient cohort of transferred patients and the patients who were recruited directly at the clinic in Rostock. Patients were classified according to hip and knee revision arthroplasty. Various criteria such as comorbidities, case severity and length of stay (LOS) were compared. In addition, treatment costs based on DRG charges (Diagnosis-Related Group) were determined up to one year following the hospital stay to quantify any additional financial burden imposed by the different patient cohorts. Special attention was paid to septic revision procedures, as there is generally a higher readmission rate among patients after replacement surgery due to periprosthetic joint infection (PJI) [4].

Based on the results, the aim of this study was to discuss whether and to what extent cooperation between AC and ACmax needs to be further developed and optimized to ensure the best possible patient care. In this context, it was taken into account whether there were differences in the previously described criteria for patients transferred from certified and non-certified clinics.

## 2. Materials and Methods

In this retrospective study, the routine data of patients who underwent hip and knee arthroplasty between 2015 and 2021 were analyzed. Only patients with the procedure codes 5-821 (inspection, replacement and removal of a hip joint endoprosthesis) and 5-823 (inspection, replacement and removal of a knee joint endoprosthesis) [5] were included in the analyses. These patients were divided into two groups. The first group (n = 60) comprised transferred patients who were previously treated as inpatients in other clinics and transferred directly to our clinic or who were admitted to us within 24 h of discharge from the external facilities [6]. The second group (n = 76), also called the control group, included all patients who were directly admitted to our clinic in 2019. The year 2019 was chosen as the reference year to exclude any influence of the SARS-CoV-2 virus pandemic on inpatient procedures. Consequently, a patient cannot have been assigned to both groups.

For the comparison of comorbidities between the two groups, different comorbidity measures were used. The “Original Charlson Comorbidity Index” (CCI), developed in 1987, includes 19 weighted comorbidities that influence the 1-year mortality [7]. The highest possible CCI is 29. With the “Updated Charlson Comorbidity Index”, a more recent version of the original index was established in 2011. In that version, only 12 comorbidities still affect patient outcomes, with a maximum index of 24 [8]. The “Van Walraven Elixhauser Index” reflects a scoring system of 30 comorbidities and their impact on hospital mortality. Negative effects are also possible and the index can range from −19 to +89 [9,10]. The “AHRQ Elixhauser Index” weighted the same comorbidities except for cardiac arrhythmias, with possible overall values from −32 to +99 [11]. Tables S1 and S2 summarize the four indices [12]. The indices were determined for all patients by considering the ICD codes (International Statistical Classification of Diseases and Related Health Problems) for calculation and excluding the main diagnosis [13,14]. In addition, information regarding the Case Mix Index (CMI), where higher values are associated with higher case severity and resource use [15,16], the Patient Clinical Complexity Level (PCCL), i.e., the patient-related cumulative severity of comorbidities and/or complications, ranging from 0 (no clinical complexity) to 4 (very severe clinical complexity) [17], the hospital LOS and the DRG were obtained from the hospital information system for each patient. 

All patients were followed up for one year after scheduled discharge. In the case of two-stage revision procedures, the one-year follow-up period began after the reimplantation of the endoprosthesis. The total number of readmissions per patient within the postoperative year was examined, including planned readmissions in the context of two-stage replacements. The total costs for each patient were calculated as the sum of all DRG charges. For transferred patients, only readmissions to our clinic were evaluated, while for self-recruited patients, admissions to other clinics were also taken into account to analyze the recurrence rate after surgery. Data from the German Arthroplasty Registry (EPRD) [18], the hospital’s internal information system and one-year patient surveys were used to determine the recurrence rate. All patients were followed up for one year and there was no loss to follow-up.

### 2.1. Septic Revision Arthroplasty

**Figure 1:**
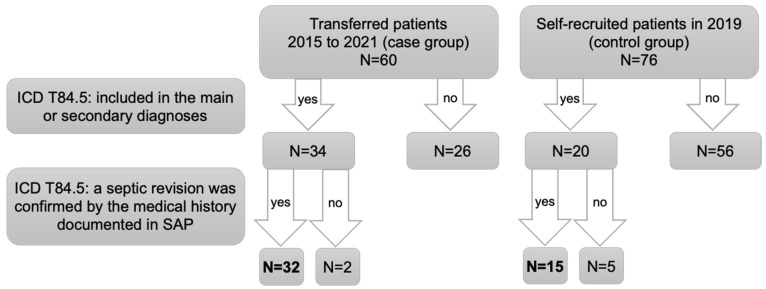
Inclusion and exclusion criteria for septic revision arthroplasty.

All patients were reviewed in terms of the cause for the revision arthroplasty to identify those for whom the revision was due to an infection of the affected joint. Identification was solely based on the record of ICD T84.5 in the main or secondary diagnoses. The diagnosis was confirmed by further medical data, leading to the definition of two further subgroups within the previously identified infection group (Figure 1).

In these group comparisons, it was examined whether a new or recurrent prosthetic joint infection occurred after infection clearance and if so, within which period. Additionally, the mortality rate was investigated and whether complications, including amputation, occurred during the clearance phase or in the follow-up period.

Two patients from the transfer group were excluded retrospectively. In one case, the infection was only observed during the surgery itself. In the other case, the cause of revision arthroplasty was identified as aseptic loosening, even though the ICD diagnosis T84.5 was documented as part of a past medical history. Of the septic revision surgeries in the control group, five had to be excluded as they were retrospectively identified as aseptic revisions (Figure 1). The one-year follow-up was also based on DRG data, one-year surveys, data from the EPRD and documentation in the hospital information system. The follow-up began at the time of discharge after the completion of infection treatment and under the condition that no readmissions for reimplantation of an arthroplasty were planned.

### 2.2. Statistical Analysis

The statistical analysis was performed using the SPSS 29.0.1.1 statistical software (IBM Deutschland GmbH, Ehningen, Germany). Initially, the parameters in the respective groups were tested separately for normal distribution using the Shapiro–Wilk test. If the parameter showed a normal distribution in both groups, the significance of the differences between the two groups was tested using *t*-tests for independent samples. If the data were not normally distributed, the Mann–Whitney U-test was performed to compare the groups. Since the majority of the data were not normally distributed, the Mann–Whitney U-test was used, unless otherwise stated. To test whether the two groups differed significantly in terms of nominal characteristics, Fisher’s exact test was used. For septic revision procedures, the odds ratio for complications during the one-year follow-up was also calculated. A significance level of *p* < 0.05 indicated significant differences between the compared groups.

## 3. Results

In total, the data of 136 patients were analyzed. These included 60 transferred patients (group 1, case group) and 76 self-recruited patients (group 2, control group).

Table 1 shows that there were no differences in gender or regarding the treated joint between groups 1 and 2. Thus, there is no bias for these two factors. However, the number of interventions due to joint infection differed significantly between the groups (*p* < 0.001).

Significant differences between the two groups were also observed for all analyzed parameters, except for age and the original Charlson Comorbidity Index (CCI) (Table 2). Transferred patients had a higher CMI, a higher PCCL, a longer hospital stay, higher DRG charges, more readmissions and more comorbidities. There were also significant correlations between comorbidity indices and surgical difficulty as well as comorbidity indices and age, but no correlations between age and surgical difficulty as assessed by CMI and PCCL (Table S3). It was taken into account that one patient among the self-recruited patients was readmitted to a clinic other than the ACmax, as revealed by EPRD tracking.

For a more detailed analysis, patient data were examined separately for the two different joints, i.e., hip and knee. The results (Table 3) show that among transferred patients, the percentage of septic revisions was significantly higher than in the patients recruited directly in the ACmax.

Significant differences between transferred and self-recruited patients with knee revision arthroplasty are shown in Table 4. As listed, CMI, PCCL and both Elixhauser indices were worse in transferred patients. These patients also had a longer stay during the first inpatient admission, longer combined total hospital stays during the one-year follow-up and, consequently, higher DRG charges since these patients were readmitted more often. There were no differences for age and both Charlson comorbidity indices.

For hip revision patients, only one significant difference was observed between groups 1 and 2 for PCCL (*p* < 0.001). While the transferred patients had a mean PCCL of 3.38 (standard deviation (SD) = 1.52), the control group was significantly less impaired with a mean PCCL of 1.78 (SD = 1.58). As there were no further significant differences for hip revision arthroplasty except for the PCCL, we refrained from presenting further hip revision arthroplasty results in detail.

It was furthermore investigated whether the patient population, including both hip and knee revision arthroplasty, transferred from EndoCert-certified hospitals (AC, n = 40) differed from the patients transferred from non-certified hospitals (n = 20) to the ACmax. However, there were no significant differences in age, CMI, PCCL, comorbidity indices, LOS, readmission rates or DRG charges in the patient population between certified and non-certified hospitals.

Since our results showed a significant difference in the proportion of septic revision arthroplasty between group 1 and group 2, we separately analyzed the patient cohorts that underwent revision arthroplasty due to PJI. Based on the ICD T84.5, 32 septic revisions were identified in the group of external transfers, while the control group included 15 septic patients (Table 5). Septic recurrence and amputation of the infected limb were only observed in transferred patients. Thus, there was a slightly increased risk of both septic recurrence and amputation (OR 1.143) in group 1, but the change was not significant (*p* = 0.291). The four observed cases were divided equally between knee and hip revisions with two cases each, resulting in a reinfection rate of 14.3% for septic hip arthroplasty and 11.1% for septic knees among the transferred patients. There were no significant differences in mortality (*p* = 0.648, OR = 0.672).

The analysis revealed that transferred patients with septic revision arthroplasty, despite being significantly younger than the patients recruited directly in the ACmax itself, were considerably more affected with regard to the PCCL (Table 5). Due to the small group sizes, no further significant differences in comorbidity indices, CMI, hospital LOS and DRG charges were detected.

## 4. Discussion

As an ACmax in Germany in the relatively sparsely inhabited state of Mecklenburg-Vorpommern, our clinic has a large catchment area with regard to patients in need of a transfer from other hospitals. Our study showed that these transferred patients required a considerable amount of additional time, care and resources. Not only did they represent an additional cost factor, but they were also more cost-intensive in direct comparison with the patients recruited by the ACmax itself. The collected clinical parameters are closely interrelated and sometimes directly influence each other, as explained below [11,19,20,21].

The classification into degrees of severity (PCCL) is intended to offer the possibility to adapt to the individual case-related cost requirements. The average PCCL of transferred patients was 3.42, and they were classified into the categories of (very) severe complications and comorbidities, while the complications and comorbidities of the self-recruited patients of the ACmax were classified as mild to moderately severe with an average PCCL of 1.67 [17]. Bayliss-McCulloch et al. [22] showed a positive correlation of the PCCL with the LOS, the mean daily costs and the total costs. However, the strength of the correlation was only classified as low (LOS, total costs) or moderate (mean daily costs) [22,23]. According to our findings, transferred patients tended to be younger on average, but with a higher PCCL. This paradox reflects the inclusion of especially difficult cases in the transfer group.

The CMI reflects average case severity and resource utilization [15,16]. The case-based hospital statistics from 2018 showed an average CMI of 1.11 for all patients in Mecklenburg-Vorpommern [24]. Generally, hip and knee revision arthroplasty already requires high resource utilization, as shown by the average CMI values of 5.87 for the transferred and 3.50 for the self-recruited patients. The higher CMI of the externally transferred patients indicated that these patients require even more intensive care, thus supporting the hypothesis that this is leading to an increased workload in the ACmax. 

Similar effects were observed when analyzing the comorbidities. For the assessment of comorbidities, the Charlson indices as well as the Elixhauser indices were each used in two variants [7,8,9,10,11]. These indices serve as theoretical benchmarks for quantifying the complexity of a patient’s case but can ultimately result in different treatment requirements depending on the severity of the comorbidities. The higher value of the original CCI and the updated CCI for the transferred patients indicated a comparatively increased number or more severely weighted comorbidities in addition to the main disease, which affects overall survival. Based on the ten-year survival probability predicted by Charlson et al. in 1987 [7], the transferred patients are likely to have a lower survival rate compared to the self-recruited patients. Due to increased life expectancy and changing disease profiles, the timeliness of the weighting of individual comorbidities has been questioned. The updated CCI offers a more advanced calculation method [8]. However, Quan et al. [8] only considered in-hospital mortality and did not update the survival prognoses over ten years [7]. Both indices showed differences in hospital mortality as well as a consistent association with higher mortality rates [8], thus predicting a higher mortality rate for the transferred patients. A US study from 2014 [20] showed that in addition to mortality, the CCI influences the number of readmissions after arthroplasty. Each additional point in the CCI increases the risk of readmission by 0.45%. Johnson et al. [21] confirmed that a higher CCI is associated with longer hospital stays and higher average case costs. Our own results showed that higher CCI values in the case group were associated with a statistically significant higher number of readmissions. This might be due to the higher percentage of septic revisions among transferred patients since septic revisions are associated with a higher readmission rate [4,25]. Elixhauser et al. [9] and van Walraven et al. [10] showed that a higher Elixhauser Index is associated with longer hospital stays and an increased risk of death, as well as higher case costs per patient and an increased need for medical care. Transferred patients showed significantly higher Elixhauser indices, indicating a longer LOS and higher costs. Further studies such as Hinton et al. [19] confirmed the predictive power of the Elixhauser Index for costs and LOS in primary knee arthroplasty. Goltz et al. [26] showed that a higher Elixhauser Index is associated with an increased 90-day readmission risk. This is concurrent with our data, as the transferred patients not only showed higher AHRQ and Van Walraven Elixhauser indices but also longer inpatient stays, a higher readmission rate within one year after surgery and consequently, an overall increased hospital LOS up to one year after surgery. 

While the described overall results were confirmed for the subgroup of patients with knee revision arthroplasties only, there were no significant differences for hip revision arthroplasty except for the PCCL. A larger study is necessary to clarify whether the lack of significance is due to the small group sizes.

The cost between transferred patients and self-recruited patients differed significantly, both during the first inpatient admission and up to one year after surgery. Many different factors are included in the cost calculation, which is why a generalization or precise cost prediction for hip and knee revision arthroplasty is only possible to a limited extent. Even if the DRG code is identical, the final costs may differ due to additional services or exceeding the maximum as well as undercutting the minimum LOS [27]. Furthermore, a comparison to other studies is only possible to a limited extent due to different inclusion criteria, lack of exact costs, different currencies and healthcare systems, as well as increasing base rates [28,29]. Studies from France [30] and Germany [29,31,32] provided insights into the potential costs of hip and knee replacement surgery. In general, septic replacement procedures are associated with higher costs [29,30,33]. The results of our study are concurrent with the literature, but we did not differentiate between septic and aseptic in the current analysis. In an investigation by Weber et al. [31], the costs that were determined for hip and knee revision arthroplasty were lower compared to the costs in our study; however, the proportion of septic revisions was also considerably lower compared to our data. When differentiating between knee and hip revisions, it was found that knee revision arthroplasty was significantly more expensive for transferred patients compared to directly recruited patients.

The diagnosis-related tariffs only take the average costs into account, but the actual costs may deviate from this, especially for complex cases. Studies showed deficits or (scarce) coverage in the billing of (septic) revision arthroplasties [29,31,32,34,35]. The transfer of high-risk revision surgeries to maximum-care arthroplasty centers is important for patient care but requires appropriate reimbursement to avoid financial loss to the admitting hospital. So far, neither ACmax nor AC has received more financial resources from the Ministry of Health compared to non-certified hospitals.

There were some extreme cases in our study. In one case, a transferred patient was hospitalized for a total of 152 days considering the initial hospital stay and all readmissions within the one-year follow-up period. A DRG charge of EUR 108,480.08 was listed for the most cost-intensive patient. These examples illustrate the enormous time and cost expenditure for an ACmax due to the assigned cases.

We examined whether there were differences in the analyzed parameters if patients were transferred from certified and non-certified clinics. It was expected that transfers from certified clinics would demand even higher resource utilization, as ACs are already specialized hospitals [3] with standardized structures and processes in arthroplasty care. For this reason, only the transfer of clinically more complex cases should be necessary, which would be reflected in higher CCI, Elixhauser, CMI, PCCL, LOS, readmissions and DRG charges for those transferred from certified hospitals. However, only the updated CCI showed higher values for transferred patients from certified clinics. Further differences were not detected, probably due to the small sample sizes. In this context, we additionally examined the structured quality reports of the certified AC in order to calculate the revision rate, which results from the relative ratio of revision surgeries to the total number of surgeries [36,37]. It was found that in the years included in this study, in the referring Acs, only 6.9% of all arthroplasty surgeries were revision arthroplasties. This result suggests that the surrounding ACs primarily ensure elective arthroplasty and therefore no difference could be demonstrated compared to the non-certified clinics, which had a comparable average revision rate of 7.3%.

Septic revision arthroplasty represents a particular challenge for hospitals [38,39,40,41]. According to the EPRD, 16.7% of hip revisions and 15.0% of knee revisions were performed due to PJI in 2021 [42]. In the case group, 53.3% were septic revisions, while in the control group, only 19.7% had undergone septic revision arthroplasty. The transferred patients with PJI were categorized as (very) severe complications and comorbidities based on their PCCL [17]. Paradoxically, transfer patients were significantly younger, whereas increasing PCCL values would rather be expected in older patients. A study with a larger sample size is necessary to conclude that these are the only relevant differences between the subgroups.

Apart from the lower proportion of septic revisions, the self-recruited patients also showed no septic recurrences at our clinic within one year. In contrast, 12.5% of the septic transfers (hip: 14.3%, knee: 11.1%) were revised again after an average of 240 days due to infection. The risk of recurrence was increased by a factor of 1.143 without reaching significance. A meta-analysis from 2012 revealed an average reinfection rate of 11.3% for septic revisions of hip arthroplasties. One-stage septic replacements had higher reinfection rates (13.1%) compared to two-stage replacements (10.4%) [43]. Successful rehabilitations were achieved in 83% or 76% (including deaths) of two-stage operations with an average follow-up interval of 53 months [44]. Another study from 2016 [45] reported a 93% eradication success rate for one-stage revisions of infected knee arthroplasties over ten years. Our calculated reinfection rate of transferred patients is in agreement with the data from these studies. The 100% eradication success of the septic cases in the group of self-recruited patients indicates high-quality treatment, but the short follow-up period compared to other studies should be considered. Additionally, the transferred patient fared worse with regard to treatment failure as 12.5% of the cases of transferred patients with septic revision arthroplasty resulted in amputations. Data on the frequency of amputation due to joint infection after arthroplasty are limited, although this procedure is generally considered rare [46]. A previous study on the outcome after treatment of PJI in our ACmax showed an overall disarticulation rate of 6.9% for septic revisions [47].

The mortality rates in the two groups of septic revisions did not differ significantly at 9.4% and 13.3%. Retrospectively, it was not possible to attribute the outcome completely to the infected arthroplasty. Several studies reported higher mortality after septic revisions [48,49]. The 1-year mortality rate after septic revisions was 5.3 times higher than after aseptic revisions [48] and the 1-year mortality risk was 3.1 times higher than the national age-adjusted mortality [49].

The increased socioeconomic burden due to septic revisions is also reflected by the higher costs of arthroplasty treatment compared to aseptic inpatient stays [29,30,33]. A study on the treatment of periprosthetic hip infections at the Orthopedic Clinic and Policlinic Rostock in 2012 [32] determined average costs of EUR 29,331.36 and an average LOS of 52.7 days per patient. This illustrates the enormous time expenditure and is comparable to the LOS observed in this study. In general, septic procedures are associated with significantly longer hospital stays compared to aseptic arthroplasties [33]. The costs of treating a periprosthetic joint infection are highly variable, and a prediction is often not possible due to the many influencing factors. However, higher costs are often associated with increased values of clinical parameters such as PCCL, CCI or the Elixhauser Index. These indices might therefore function as predictors of the expected case costs [9,19,21,22,50].

## 5. Limitations

1. With regard to the reinfection rate after septic revision surgery, the follow-up period of one year is comparatively short.
2. This study examined the additional effort for transferred patients compared to self-recruited patients. Further work should investigate whether the tariffs received by the ACmax for the transferred patients cover the costs of the treatment or result in a loss for the ACmax.
3. This study assumed that documented diagnostic codes provided comprehensive information regarding the entire medical data of the patient.
4. This study was based on data from an ACmax in a rural state with a wide catchment area.

## 6. Conclusions

Our results support the necessity of cooperation between ACs and ACmax to enhance patient safety. However, this cooperation requires considerable resources on the part of the ACmax. In the interest of the best possible care, already coordinated agreements on radiological imaging, laboratory tests and clinical assessments should be added to the cooperation agreements in order to avoid loss of information and optimize the transfer process. To promote interactive cooperation, the treatment outcomes should be reevaluated between the centers, such as within the framework of quality circles or joint complication meetings. Further analyses should examine whether more favorable results can be achieved through improved coordinated transfer algorithms and, in particular, earlier transfers in cases of septic complications.

## Figures and Tables

**Table 1:** Demographic data of externally transferred patients and patients recruited in the ACmax.

		Transferred Patients Group 1 (n = 60)	Self-Recruited Patients Group 2 (n = 76)	Fisher’s Exact Test (*p*-Value)
Gender	Male	22 (36.67%)	27 (35.53%)	1.000
	Female	38 (63.33%)	49 (64.47%)	
Joint	Hip	34 (56.67%)	45 (59.21%)	0.861
	Knee	26 (43.33%)	31 (40.79%)	
Septic revision arthroplasty	Yes	32 (53.33%)	15 (19.74%)	<0.001
	No	28 (46.67%)	61 (80.26%)	

**Table 2:** Comparison of externally transferred patients (group 1) with patients recruited in the ACmax (Group 2).

		Median (Minimum; Maximum)	Mean Value (Standard Deviation)	*p*-Value
Age at surgery (years)	Group 1	72.50 (43; 91)	71.80 (10.11)	0.184
	Group 2	76.00 (40; 91)	73.76 (9.67)	
Case Mix Index (CMI)	Group 1	3.88 (1.46; 29.59)	5.87 (5.62)	<0.001
	Group 2	3.01 (1.69; 12.58)	3.50 (1.64)	
Patient Clinical Complexity Level (PCCL)	Group 1	4.00 (0; 6)	3.42 (1.44)	<0.001
	Group 2	1.00 (0; 5)	1.67 (1.57)	
Original Charlson Comorbidity Index	Group 1	1.00 (0; 11)	1.65 (2.06)	0.092
	Group 2	1.00 (0; 8)	1.08 (1.51)	
Updated Charlson Comorbidity Index	Group 1	1.00 (0; 10)	1.35 (1.90)	0.049
	Group 2	0.00 (0; 6)	0.78 (1.28)	
AHRQ Elixhauser Index	Group 1	11.00 (−6; 43)	13.45 (12.38)	0.005
	Group 2	10.00 (−11; 43)	7.64 (9.22)	
Van Walraven Elixhauser Index	Group 1	9.00 (−4; 35)	9.43 (9.01)	0.009
	Group 2	5.00 (−7; 34)	5.64 (6.66)	
Length of hospital stay (LOS) at first inpatient admission (days)	Group 1	20.00 (9; 148)	28.12 (23.60)	0.004
	Group 2	15.00 (7; 42)	18.08 (7.83)	
Diagnosis-Related Group (DRG) charge per patient at first inpatient admission (Euro)	Group 1	13,238.17 (5465.41; 108,480.08)	20,430.13 (19,381.18)	<0.001
	Group 2	10,637.20 (5976.04; 44,412.57)	12,337.34 (5799.67)	
Number of inpatient readmissions	Group 1	0.00 (0; 3)	0.52 (0.79)	0.015
	Group 2	0.00 (0; 5)	0.28 (0.76)	
Total hospital LOS (days)	Group 1	28.00 (9; 152)	41.53 (36.91)	<0.001
	Group 2	15.50 (7; 105)	23.38 (18.23)	
DRG total sum per patient (Euro)	Group 1	21,338.20 (5465.41; 108,480.08)	28,922.50 (24,047.04)	<0.001
	Group 2	10,967.24 (5976.04; 65,510.51)	15,632.87 (11,565.32)	

**Table 3:** Demographic data of externally transferred patients and patients recruited in the ACmax divided due to hip and knee revision arthroplasty.

Joint			Transferred Patients	Self-Recruited Patients	Fisher’s Exact Test (*p*-Value)
Hip	n		34	45	
	Gender	Male	14 (41.18%)	16 (35.56%)	0.646
		Female	20 (58.82%)	29 (64.44%)	
	Septic revision arthroplasty	Yes	14 (41.18%)	9 (20.00%)	0.049
		No	20 (58.82%)	36 (80.00%)	
Knee	n		26	31	
	Gender	Male	8 (30.77%)	11 (35.48%)	0.782
		Female	18 (69.23%)	20 (64.52%)	
	Septic revision arthroplasty	Yes	18 (69.23%)	6 (19.35%)	<0.001
		No	8 (30.77%)	25 (80.65%)	

**Table 4:** Comparison of externally transferred patients (group 1) with patients recruited in the ACmax (group 2) for knee revision arthroplasty.

		Median (Minimum; Maximum)	Mean Value (Standard Deviation)	*p*-Value
Case Mix Index (CMI)	Group 1	4.19 (2.39; 27.22)	6.63 (6.19)	<0.001
	Group 2	3.11 (1.69; 6.35)	3.19 (1.28)	
Patient Clinical Complexity Level (PCCL)	Group 1	3.50 (0; 6)	3.46 (1.36)	<0.001
	Group 2	1.00 (0; 5)	1.52 (1.57)	
AHRQ Elixhauser Index	Group 1	11.50 (−5; 40)	14.85 (13.08)	0.005
	Group 2	5.00 (−11; 43)	5.68 (11.25)	
Van Walraven Elixhauser Index	Group 1	9.00 (−4; 28)	10.08 (9.18)	0.016
	Group 2	1.00 (−7; 34)	5.00 (8.60)	
Length of hospital stay (LOS) at first inpatient admission (days)	Group 1	22.50 (10; 148)	32.62 (28.50)	<0.001
	Group 2	15.00 (7; 37)	16.00 (6.45)	
Diagnosis-Related Group (DRG) charge per patient at first inpatient admission (Euro)	Group 1	14,687.95 (8256.85; 89,215.94)	22,586.27 (20,304.29)	<0.001
	Group 2	10,967.24 (5976.04; 22,400.43)	11,259.65 (4506.06)	
Number of inpatient readmissions	Group 1	0.50 (0; 3)	0.65 (0.80)	0.002
	Group 2	0.00 (0; 1)	0.13 (0.34)	
Total hospital LOS (days)	Group 1	37.00 (10; 148)	49.50 (41.62)	<0.001
	Group 2	15.00 (7; 52)	18.32 (10.02)	
DRG total sum per patient (Euro)	Group 1	26,938.54 (8256.85; 94,261.96)	34,836.35 (24,508.22)	<0.001
	Group 2	10,967.24 (5976.04; 27,426.93)	12,900.80 (6789.74)	

**Table 5:** Comparison of externally transferred patients and patients recruited in the ACmax with septic revision arthroplasty.

		Transferred Patients (n = 32)	Self-Recruited Patients (n = 15)	*p*-Value	Odds Ratio
Gender	Male	14 (43.75%)	10 (66.67%)	0.212 ^#^	/
	Female	18 (56.25%)	5 (33.33%)		
Joint	Hip	14 (43.75%)	9 (60.00%)	0.359 ^#^	/
	Knee	18 (56.25%)	6 (40.00%)		
Septic recurrence	Yes	4 (12.50%)	0 (0.00%)	0.291 ^#^	1.143
Average time until recurrence	Days	240	/	/	/
Amputation	Yes	4 (12.50%)	0 (0.00%)	0.291 ^#^	1.143
Mortality	Yes	3 (9.38%)	2 (13.33%)	0.648 ^#^	0.672
Age	Mean ± SD	66.22 ± 8.93	74.53 ± 8.07	0.004 *	/
PCCL	Mean ± SD	3.94 ± 1.05	2.53 ± 1.55	0.003 ^§^	/

^#^ = Fisher’s exact test, ^§^ = Mann–Whitney U test, * = *t*-Test.

## Data Availability

The data presented in this study are available upon request from the corresponding author. The data are not publicly available but can be obtained from the Department of Clinical Research at the Orthopedic Department of the University Medicine Rostock if required.

## Supplementary Materials

The following supporting information can be downloaded at: https://www.mdpi.com/article/10.3390/healthcare12181869/s1, Table S1: Original and Updated Charlson Comorbidity Index. Table S2: Elixhauser Index by van Walraven and AHRQ Elixhauser Index. Table S3: Correlation of comorbidity indices, age and surgical difficulty.
