# Supplementary material for: Comparison of Externally Transferred and Self-Recruited Patients with Hip and Knee Revision Arthroplasty at a Certified Maximum-Care Arthroplasty Center (ACmax)
Source: Healthcare (Basel). 2024 Sep 18;12(18):1869. doi: 10.3390/healthcare12181869 (PMC11431506; doi:10.3390/healthcare12181869)
Supplement: Supplementary file 1 [file healthcare-12-01869-s001.zip › healthcare-3190089-supplementary.pdf]

Table_S1: Original and Updated Charlson Comorbidity Index

Comorbidity	ICD-10 Codes	Original Charlson Comorbidity Index	Updated Charlson Comorbidity Index
Myocardial infarction	I21.x, I22.x, I25.2	1	0
Congestive Heart Failure	I09.9, I11.0, I13.0, I13.2, I25.5, I42.0, I42.5-I42.9, I43.x, I50.x, P29.0	1	2
Peripheral vascular disease	I70.x, I71.x, I73.1, I73.8, I73.9, I77.1, I79.0, I79.2, K55.1, K55.8, K55.9, Z95.8, Z95.9	1	0
Cerebrovascular disease	G45.x, G46.x, H34.0, I60.x-I69.x	1	0
Dementia	F00.x-F03.x, F05.1, G30.x, G31.1	1	2
Chronic pulmonary disease	I27.8, I27.9, J40.x-J47.x, J60.x-J67.x, J68.4, J70.1, J70.3	1	1
Rheumatic disease	M05.x, M06.x, M31.5, M32.x-M34.x, M35.1, M35.3, M36.0	1	1
Peptic ulcer disease	K25.x-K28.x	1	0
Mild liver disease	B18.x, K70.0-K70.3, K70.9, K71.3-K71.5, K71.7, K73.x, K74.x, K76.0, K76.2-K76.4, K76.8, K76.9, Z94.4	1	2
Diabetes without chronic complication	E10.0, E10.1, E10.6, E10.8, E10.9, E11.0, E11.1, E11.6, E11.8, E11.9, E12.0, E12.1, E12.6, E12.8, E12.9, E13.0, E13.1, E13.6, E13.8, E13.9, E14.0, E14.1, E14.6, E14.8, E14.9	1	0
Diabetes with chronic complication	E10.2-E10.5, E10.7, E11.2-E11.5, E11.7, E12.2-E12.5, E12.7, E13.2-E13.5, E13.7, E14.2-E14.5, E14.7	2	1
Hemiplegia or paraplegia	G04.1, G11.4, G80.1, G80.2, G81.x, G82.x, G83.0-G83.4, G83.9	2	2
Renal disease	I12.0, I13.1, N03.2-N03.7, N05.2-N05.7, N18.x, N19.x, N25.0, Z49.0-Z49.2, Z94.0, Z99.2	2	1
Any malignancy, including lymphoma and leukemia, except malignant neoplasm of skin	C00.x-C26.x, C30.x-C34.x, C37.x-C41.x, C43.x, C45.x-C58.x, C60.x-C76.x, C81.x-C85.x, C88.x, C90.x-C97.x	2	2
Moderate or severe liver disease	I85.0, I85.9, I86.4, I98.2, K70.4, K71.1, K72.1, K72.9, K76.5, K76.6, K76.7	3	4
Metastatic solid tumor	C77.x-C80.x	6	6
AIDS/HIV	B20.x-B22.x, B24.x	6	4

Own presentation based on: [7, 8, 12]

Table_S2: Elixhauser Index by van Walraven and AHRQ Elixhauser Index

Comorbidity	ICD-10 Codes	Elixhauser Index by van Walraven	AHRQ Elixhauser Index
Congestive heart failure	I09.9, I11.0, I13.0, I13.2, I25.5, I42.0, I42.5-I42.9, I43.x, I50.x, P29.0	7	9
Cardiac arrhythmias	I44.1-I44.3, I45.6, I45.9, I47.x-I49.x, R00.0, R00.1, R00.8, T82.1, Z45.0, Z95.0	5	/
Valvular disease	A52.0, I05.x-I08.x, I09.1, I09.8, I34.x-I39.x, Q23.0-Q23.3, Z95.2-Z95.4	-1	0
Pulmonary circulation disorders	I26.x, I27.x, I28.0, I28.8, I28.9	4	6
Peripheral vascular disorders	I70.x, I71.x, I73.1, I73.8, I73.9, I77.1, I79.0, I79.2, K55.1, K55.8, K55.9, Z95.8, Z95.9	2	3
Hypertension, uncomplicated	I10.x	0	-1
Hypertension, complicated	I11.x-I13.x, I15.x	0	-1
Paralysis	G04.1, G11.4, G80.1, G80.2, G81.x, G82.x, G83.0-G83.4, G83.9	7	5
Other neurological disorders	G10.x-G13.x, G20.x-G22.x, G25.4, G25.5, G31.2, G31.8, G31.9, G32.x, G35.x-G37.x, G40.x, G41.x, G93.1, G93.4, R47.0, R56.x	6	5
Chronic pulmonary disease	I27.8, I27.9, J40.x-J47.x, J60.x-J67.x, J68.4, J70.1, J70.3	3	3
Diabetes, uncomplicated	E10.0, E10.1, E10.9, E11.0, E11.1, E11.9, E12.0, E12.1, E12.9, E13.0, E13.1, E13.9, E14.0, E14.1, E14.9	0	0
Diabetes, complicated	E10.2-E10.8, E11.2-E11.8, E12.2-E12.8, E13.2-E13.8, E14.2-E14.8	0	-3
Hypothyroidism	E00.x-E03.x, E89.0	0	0
Renal failure	I12.0, I13.1, N18.x, N19.x, N25.0, Z49.0-Z49.2, Z94.0, Z99.2	5	6
Liver disease	B18.x, I85.x, I86.4, I98.2, K70.x, K71.1, K71.3-K71.5, K71.7, K72.x-K74.x, K76.0, K76.2-K76.9, Z94.4	11	4
Peptic ulcer disease excluding bleeding	K25.7, K25.9, K26.7, K26.9, K27.7, K27.9, K28.7, K28.9	0	0
AIDS/HIV	B20.x-B22.x, B24.x	0	0
Lymphoma	C81.x-C85.x, C88.x, C96.x, C90.0, C90.2	9	6
Metastatic cancer	C77.x-C80.x	12	14
Solid tumor without metastasis	C00.x-C26.x, C30.x-C34.x, C37.x-C41.x, C43.x, C45.x-C58.x, C60.x-C76.x, C97.x	4	7

Rheumatoid arthritis/ collagen vascular diseases	L94.0, L94.1, L94.3, M05.x, M06.x, M08.x, M12.0, M12.3, M30.x, M31.0-M31.3, M32.x-M35.x, M45.x, M46.1, M46.8, M46.9	0	0
Coagulopathy	D65-D68.x, D69.1, D69.3-D69.6	3	11
Obesity	E66.x	-4	-5
Weight loss	E40.x-E46.x, R63.4, R64	6	9
Fluid and electrolyte disorders	E22.2, E86.x, E87.x	5	11
Blood loss anemia	D50.0	-2	-3
Deficiency anemia	D50.8, D50.9, D51.x-D53.x	-2	-2
Alcohol abuse	F10, E52, G62.1, I42.6, K29.2, K70.0, K70.3, K70.9, T51.x, Z50.2, Z71.4, Z72.1	0	-1
Drug abuse	F11.x-F16.x, F18.x, F19.x, Z71.5, Z72.2	-7	-7
Psychoses	F20.x, F22.x-F25.x, F28.x, F29.x, F30.2, F31.2, F31.5	0	-5
Depression	F20.4, F31.3-F31.5, F32.x, F33.x, F34.1, F41.2, F43.2	-3	-5

Own presentation based on: [9, 10, 11, 12]

Table_S3: Correlation of comorbidity indices, age and surgical difficulty

			Age	Original Charlson Weight Score	Updated Charlson Weight Score	AHRQ Elixhauser Score	Van Walraven Elixhauser Score
Patients recruited in the ACmax	Age	correlation coefficient r	1	0.266*	0.325*	0.268*	0.367**
		p-value		0.040	0.011	0.038	0.004
		N	60	60	60	60	60
	CM-Index	correlation coefficient r	-0.249	0.342**	0.321*	0.442**	0.309*
		p-value	0.055	0.007	0.012	<0.001	0.016
		N	60	60	60	60	60
	DRG Patient Clinical Complexity Level	correlation coefficient r	-0.038	0.302*	0.372**	0.523**	0.463**
		p-value	0.771	0.019	0.003	<0.001	<0.001
		N	60	60	60	60	60
Transferred patients	Age	correlation coefficient r	1	0.258*	0.231*	0.348**	0.335**
		p-value		0.024	0.045	0.002	0.003
		N	76	76	76	76	76
	CM-Index	correlation coefficient r	-0.097	0.121	0.326**	0.398**	0.408**
		p-value	0.403	0.298	0.004	<0.001	<0.001
		N	76	76	76	76	76
	DRG Patient Clinical Complexity Level	correlation coefficient r	0.203	0.393**	0.506**	0.559**	0.570**
		p-value	0.079	<0.001	<0.001	<0.001	<0.001
		N	76	76	76	76	76
	*p-value < 0.05						
	**p-value < 0.01						
